# Rising sun or strangled in the cradle? A narrative review of near-infrared fluorescence imaging-guided surgery for pancreatic tumors

**DOI:** 10.1097/JS9.0000000000001676

**Published:** 2024-05-20

**Authors:** Kang Chen, Xiong Teng, Ning Zhou, Wei Cheng

**Affiliations:** a Department of Hepatobiliary Surgery, Hunan Provincial People’s Hospital (The First Affiliated Hospital of Hunan Normal University), Changsha; b Department of Hepatobiliary Surgery, The First Affiliated Hospital of Guangxi Medical University, Nanning, Guangxi Zhuang Autonomous Region, People’s Republic of China

**Keywords:** fluorescence imaging, indocyanine green, methylene blue, pancreatic ductal adenocarcinoma, pancreatic neuroendocrine tumors

## Abstract

Near-infrared fluorescence (NIRF)-guided surgical navigation has become a promising and effective detection method in pancreatic tumor surgery. The imaging technique has gradually transitioned from the NIR-I region to the NIR-II region. Real-time assessment of the tumor boundary and determination of the ideal resection plane are essential for preserving the pancreatic parenchyma and its secretory functions. However, since the pancreatic parenchyma has a less rich blood supply than the liver, the application of contrast agents in pancreatic tumor surgery is still in its infancy. The application of indocyanine green (ICG) and methylene blue (MB) in intraoperative NIRF imaging of pancreatic tumors has become more mature, but due to the characteristics of nonspecific imaging, the imaging efficiency and depth need to be improved. Many tumor-specific imaging agents have been designed, but most of them have not gone past animal trials because of their high development and imaging costs, biotoxicity, and other limitations. In this article, we review recent reports of ICG, MB, and newly developed contrast agents and imaging devices. We focus on the current status and new developments in the application of these contrast agents and summarize the current clinical and preclinical studies on specific contrast agents. We synthesize relevant reports to discuss the difficulties and prospects of the application of fluorescent imaging agents in pancreatic tumors. We hope that reviewing previous studies and the current progress on contrast imaging technology will provide new perspectives for its future application and development in pancreatic tumor surgery, which should translate into better patient prognoses. The manuscript was written according to the Scale for the Assessment of Narrative Review Articles (SANRA).

## Introduction

Highlights

- This article reviews indocyanine green (ICG), methylene blue (MB), and newly developed contrast agents and imaging devices in recent years. We focus on the current status and new developments in the application and summarize the current clinical and preclinical studies on contrast agents.
- Compared with other targeted contrast agents, ICG is still likely to be the safest and most widely used fluorescent contrast agent in pancreatic surgery in the future.
- Tailoring drug administration according to individual patient differences and adjusting drug concentration and injection time window offer hope for improving fluorescence visualization of pancreatic tumors.
- In the future, the development of pancreatic tumor-specific targeted contrast agents and imaging devices is promising to improve the rate of pancreatic tumor visualization, but in-vivo stability, safety, and cost of medication need to be further evaluated.
- Intraoperative near-infrared fluorescence imaging is effective in detecting pancreatic tumors, metastatic lymph nodes, and metastases, but the impact on long-term prognosis remains to be investigated with high quality and large sample size.

Many studies^1–3^ have reported the powerful effectiveness of indocyanine green (ICG) for detecting tumors on the surface of the liver as well as for surgical procedures, especially for identifying unknown preoperative lesions, but its prospects for application in pancreatic surgery are unknown.

In the enucleation (EN) of pancreatic tumors, only the tumor is removed, preserving the pancreatic parenchyma and other organs^4^. Compared with standard pancreaticoduodenectomy, pancreatectomy, and other common resection surgeries, enucleation has the advantage of not affecting pancreatic endocrine or exocrine function^5^. Therefore, evaluating tumor boundaries in real time and determining the ideal resection plane during pancreatic resection are crucial. Owing to its low cost and few side effects, near-infrared fluorescence (NIRF) imaging technology based on ICG or methylene blue (MB) can provide valuable real-time intraoperative information through simple methods and can thus provide a reference for tumor localization and resection plane determination^6^. Although ICG and MB fluorescence are used in pancreatic surgery, their specific limitations must be considered, including the low penetration depth (up to 10 mm) of the fluorescence and the low reliability in identifying pancreatitis^1,7^. Unlike ICG or MB, specific targeted fluorescent tracers can be used to specifically visualize pancreatic tumors with improved visualization depth and resolution^8^, so the preparation and application of specific targeted fluorescent tracers has become a hot research topic in recent years^9^.

The application of fluorescence-guided navigation methods for pancreatic tumor surgery has focused mainly on pancreatic cancer^10^ and pancreatic neuroendocrine tumors (PanNETs)^11^. Due to the lack of specific fluorescent contrast agents for targeting pancreatic tumors, there is still a lack of evidence on whether fluorescence-guided navigation methods for pancreatic tumor surgery are superior to traditional surgical methods. In this article, current hot topics in fluorescence-guided pancreatic surgery are interpreted based on the authors’ experience with the aim of providing a reference for fluorescence-guided navigation methods in pancreatic tumor surgery.

## Method

This narrative review was written following the Scale for the Assessment of Narrative Review Articles (SANRA) quality criteria^12^. A literature search of the PubMed database was conducted from September 2023 to October 2023 by two independent reviewers. The search terms “near-infrared region” (NIR), “fluorescence imaging” (FRI), “real-time intraoperative” (RTI), “indocyanine green” (ICG), “methylene blue” (MB), “pancreatic tumors” and “pancreatic ductal adenocarcinoma” (PDAC), and “pancreatic neuroendocrine tumors” (PanNETs) were used with Medical Subject Headings (MeSH). Manual searches were also performed by scrutinizing the reference lists of original articles, meta-analyses, and reviews.

The inclusion criteria for studies were any types of fluorescent contrast agents combined with NIRF imaging in surgery for PDAC, PanNETs, and other pancreatic tumors, as well as available literature on previously conducted and ongoing clinical and preclinical trials with fluorescent tracers. Articles published in languages other than English were excluded. For each study, we report the last name of the first author, year of publication, region/country, sample size, fluorophore, mechanism, clearance, half-life, recommended injection dose and interval before surgery, advantages and disadvantages, and fluorescence imaging devices. The data are presented in the manuscript and are summarized in Table 1.

**Table 1:** Clinical and preclinical studies evaluating targeted molecular imaging of PDAC.

Target	Fluorophore/tracer	Type of study number of cases included	Excitation wavelength (nm)	Clearance and primary clearance organ	Half-life	Recommended injection dose	Infusion-imaging window	Advantages and disadvantages	Result of visualization	Commonly used imaging devices
–	ICG	–	NIRF 820–830	>500 ml/min/m^2^ Liver	2.5–3 min	5 mg or less once 5 mg*5 times	>1 min	Safe, low cost and easy to use Non-specificity, false-positive possibility, shallow-imaging depth (5–10 mm), iodine sensitization possibility	Improved intraoperative visualization and real-time assessment of tumor outcome	Photodynamic eye, Pinpoint. Spy-Phy, Fluobeam, and FloNavi System
–	MB	–	NIRF 688 nm	Largely unmetabolized excreted in the urine	5–6 h	1–2 mg/kg	Intraoperative administration	Safe, lower cost, easy-to-use Less tissue penetration, more autofluorescence in background tissue	Improved resolution of pancreatic neuroendocrine tumors	Spy-Phy, Fluobeam, Spy Pinpoint, Infrared 800, Spy Elite
CEA	SGM-101 BM-104-anti-CEA^13^	Prospective (phase II) 12 resectable PDAC patients (5, 7.5, 10 mg)	NIRF 700 nm	NA Liver	NA	7.5 mg	48 or 96 h 96 h is more effective, injection maintenance time is 30 min	Intraoperative detection of primary PDAC and metastatic tumors is effective and safe. The fluorescence signal is greatly affected by the coverage of blood and tissue and is less effective in detecting deep tumors and areas with blood coverage	All 11 primary tumors were visualized, and the signal was consistent with CEA expression. The average TBR was 1.6 for primary tumors and 1.7 for metastatic lesions	Artemis and Spectrum fluorescence imaging systems (Quest Medical Imaging, Middenmeer, the Netherlands)
CEA	Alexa Fluor 488-anti-CEA^14^	In-vivo preclinical target validation in nude mouse model Subcutaneous (ASPC-1, BxPC-3, CFPAC, Panc-1, Capan-1) N=3 Orthotopic (BxPC-3) N=3	Fluorescence imaging 500 nm	NA NA	NA	75 μg	After 30 min, 1, 2, 6, 8, 24, 48, 192, and 360 h Best results at 23 h after injection	Compared to Oregon Green, it has a stronger and more stable fluorescence signal, especially in tumors with high CEA expression, lower background fluorescence, and minimal photobleaching effects. CEA-negative patients may not be visualized, and false positives may occur when some normal tissues express CEA; biosafety is not validated	Better visualization of primary pancreatic cancer tumors and peritoneal metastases compared to Oregon Green. Improved identification of residual tumor tissue at resection in mouse models. TBR data missing	Olympus OV-100 Small Animal Imaging System (Olympus Corp, Tokyo, Japan)
CEA	Alexa Fluor 488-anti-CEA^15^	In vivo, preclinical CEA+ FGS in nude mouse model Orthotopic PDAC mouse model (BxPC-3) N=73	Fluorescence imaging 500 nm	NA NA	NA	75 μg	24 h	Fluorescence-guided surgery (FGS) has higher cure rates and longer disease-free survival (DFS) and overall survival (OS) compared to BLS. FGS may improve surgical treatment for pancreatic cancer. Biosafety is not validated	FGS: 92% (23/25) BLS: 45.5% (10/22) 1-year OS FGS: 0% (0/22) BLS: 28% (7/25) TBR data missing	FGS: Olympus OV-100 Small Animal Imaging System (Olympus Corp, Tokyo, Japan) BLS: MVX-10 long-working distance microscope (Olympus)
CEA	hM5AIR800-anti-CEA^16^	BxPC-3-GFP nude mice, in-vitro/in-vivo preclinical probe construction and target validation in mouse model N=18	NIRF 800 nm	NA NA	NA	75 μg	After 6, 12, 24, 48, 72 h Best results at 48 h after injection	Ideal penetration depth and less autofluorescence in background tissue Susceptible to hepatic accumulation and may not be effective in identifying liver metastases	TBR 16.6 at 48 h.	Maestro CRI imaging system (Perkin Elmer, Waltham, MA, USA)
EGFR/VEGF165	Bi50-IRdye800^17^	Mice bearing BxPC-3 In-vitro/in-vivo preclinical probe construction and target validation in mouse model	NIRF 800 nm	NA Kidney	Phase1 1.34±0.34 h Phase 24.47 ±1.04 h	150 μl of 0.02 mmol	8 h	Dual targeting of EGFR/VEGF and “multistage” targeting increased the targeting of PDAC by the fusion protein Bi50 (dual targeting of blood vessels and tumor parenchyma), with better intra-tumoral penetration and accumulation. Need further clinical application verification and contraindicated for those with poor hepatic and renal function	The TBR peak of Bi50 was 4.32 ± 0.1	Fibered confocal 166 fluorescence microscopy (CFL) imaging system (Cellvizio, Mauna Kea Technologies, USA)
EGFR	Panitumumab-IRDYE800CW^18^	Prospective (phase I) 11 PDAC patients	NIRF 800 nm	NA Liver	Dose-dependent Average half-life is about 28 h	50 mg shows best results, no limit on the maximum dose	2–5 days	Improved accuracy and visualization of detection, no serious adverse effects were seen. May produce high background signal and hepatotoxic effects. False positives possibility, and fails to clarify the effect of chemotherapy and radiotherapy on fluorescence	Primary tumor TBR: 25 mg: 3.0 (±0.5) 50 mg: 4.0 (±0.6) 75 mg: 3.7 (±0.4) Sensitivity 90.3% (84.5–94.2) Specificity 74.5% (65.1–82.1) Good visualization of metastatic lymph nodes and small (<2 mm) peritoneal metastases	The SPY-PHI imaging platform (Novadaq, Burnaby, BC, Canada; an IRDye800CW tailored system) and the Explorer Air (SurgVision, Munich, Germany)
EGFR	CetuximabIRDye800CW, monoclonal antibody^19^	Prospective (phase I/II) 7 pancreatic tumors (5 PDAC, 2 NET)	NIRF 800 nm	NA Liver	NA	Best visualization at 50 mg	2–5 days	Visualize lymph nodes and tumors, distinguish pancreatitis from pancreatic tumors, safe and effective Insufficient cases and insufficient penetration depth (<1 cm) are not enough to capture the spatial characteristics of the tumor	NIRF identification of primary tumor 4/6 patients (67%) In-vivo TBR (50 mg) Primary tumor: 2.3 (±0.72) Tumor+LN: 6.3 (±0.82) Ex-vivo TBR (50 mg) Primary tumor: 3.4 (±0.4) Sensitivity 96.1%（92.2–98.4%) Specificity 67.0% (59.7–73.8%)	Laparoscopic optical imaging system PINPOINT 9000 modified for IRDye800 fluorescent dye imaging (Novadaq, Burnaby, Canada) and the wide-field SurgVision Explorer (SurgVision BV, ‘t Harde, the Netherlands)
VEGF-A	Bevacizumab-IRDye 800CW, monoclonal antibody^20^	Prospective (phase II) 10 suspected pancreatic tumors (PDAC, NET, periampullary, IPMN)	NIRF 800 nm	NA Liver	NA	In-vivo imaging TBR showed no significant differences; In-vitro imaging shows that as the dose increases, TBR is most obvious at 25 mg	3 days	Feasible and safe to use on patients with suspected PDAC. No serious adverse events were observed, and normal tissue could be distinguished from tumor tissue. Pancreatic tumors cannot be distinguished from inflammation. Specificity and number of patients are insufficient. In-vivo imaging failed to explore the optimal imaging dose. The clinical trial was terminated early	In-vivo tumor visualization with NIRF imaging differed per tumor type and was non-conclusive. Ex-vivo TBRs were 1.3, 1.5, and 2.5 for 4.5 mg, 10 mg, and 25 mg groups, respectively	IRDye-800CW-NHS (SurgVision BV, Groningen, the Netherlands)
Cath E	Ala-Gly-PheSer-Leu-ProAla-Gly-CysCONH2-Cy5.5^21^	In-vitro/in-vivo preclinical activatable probe construction and target validation in mouse model; Subcutaneous PDAC mouse model (MPanc96-E, CTSE+) Two groups of MPanc96-E tumor-bearing mice (n=6 per group)	NIRF 700 nm	NA NA	NA	2 nmol/100 μl PBS	Imaging at 2, 4, 8, 24, 48, and 72 h At 72 h, the abdominal background brightness decreased and the TBR increased significantly	Superior selectivity and sensitivity in imaging both *in vivo* and *in vitro*. This probe allows for the selective observation, enabling the detection of early enzymatic changes in cancer cells. Requires the presence of specific target proteases for activation and cannot directly image tumor cells	In-vivo TBR as high as 3 at 72 h Ex-vivo tumor-to-muscle 16	Small animal-dedicated optical imaging system (IVIS-200/100, Xenogen/Caliper, Mountain View, CA)
Cath E	Ala–Gly–Phe–Ser–Leu–Pro–Ala–Gly–CysCONH2-cy5.5^22^	In-vitro/ex-vivo preclinical probe construction and target expression in mouse model Orthotopic PDAC mouse model Human material PDAC tumor grafts model n=4 MDAPATC-3 MPanc96-FG30 Mpanc96-Cath E transgenic models n=10 each	NIRF 700 nm	NA NA	NA	1 nmol/100 μl PBS	48 h	Not only detects tumors and metastases, but also identifies pancreatic intraepithelial neoplasia (PanIN) lesions Nonspecific signals originating from the liver may affect imaging, and different grades of PanIN remain to be distinguished. Awaiting further verification in clinical trials	Pancreatic tumor TBR: tumor/normal pancreas 2.2 PanIn: TBR: three-fold specific signal	IVIS-100/Spectrum optical imaging systems (Xenogen/Caliper, Mountain View, California, USA)
CA19.9	Alexa Fluor 488-anti-CA19.9^23^	In-vitro/in-vivo preclinical probe construction and target validation in a mouse model; Orthotopic PDAC mouse model (CFPAC, BxPC-3, PANC-1)	Fluorescence imaging 500 nm	NA NA	NA	75 μg	24 h	Offers significant improvements in the visualization of small primary tumors, spleen, liver, and peritoneal metastases, compared to bright field imaging. Handheld device makes the imaging process convenient. Patients with CA19-9-negative tumors cannot be effectively imaged. Further validation through clinical trials is necessary to confirm	Metastases and primary small tumors that are invisible under a bright field are clearly visible under fluorescence. TBR data missing	Olympus OV100 Small Animal Imaging System (Olympus Corp., Tokyo, Japan)
CA19-9	Anti-CA19-9 antibody DyLight 650^24^	32 mice were randomly divided into 4 groups; BLS; BLS+NAC; FGS; FGS+NAC; Neoadjuvant chemotherapy bright light surgery fluorescence-guided surgery	Fluorescence imaging 650 nm	NA NA	NA	50 mg	24 h	Improved tissue penetration compared to AlexaFlour 488; microscopic tumors spreading around the primary tumor can be detected. Incorporating NAC into the study, the PDOX model helps determine individual tumor sensitivity to NAC regimens. No long-term survival data	Compared with FGS and BLS+NAC, FGS+NAC significantly reduced the frequency of metastatic recurrence in 8 mice; TBR data missing	A Mini Maglite LED PRO flashlight coupled to an excitation filter (ET 640/30X, Chroma) was used as the excitation light source. A Canon EOS 60D digital camera with an EF-S 18–55 IS lens coupled with an emission filter
Integrinαvβ3/αvβ5/αvβ6	cRGD-ZW800-1^25^	In-vivo preclinical feasibility and target validation in mouse model Orthotopic PDAC mouse model (BxPC-3)	NIRF 800 nm	Kidney Clearance: identical to basal glomerular filtration rate in mice	25 min	10 nmol, corresponding to a human equivalent dose of 63 μg/kg	4 h	Able to identify a variety of integrins on the surface of cancer cells and neovascular cells, so it is versatile for a variety of solid tumors. Not suitable for low-expressing integrins. Higher doses may need to be used to obtain adequate signal	TBR>2 PDAC (dose 1.0 \10\30 nmol)	Fluorescence-Assisted Resection and Exploration (FLARE, Curadel, LLC, MA, USA) system
Integrin αvβ6	R_0_1-MG-IRDye800^26^	In-vivo preclinical feasibility and target validation in mouse model Orthotopic PDAC mouse model (BxPC-3, MiaPaCa-2) Orthotopic PDAC transgenic mice (Pdx1-Cretg/+; KRasLSL G12D/+; Ink4a/Arf−/−)	NIRF 800 nm	NA Kidney	NA	30 μM	4 h	It can bind to the αvβ6 receptor on the surface of PDAC cells with high specificity and has a good renal clearance pathway. R01-MG and IRDye800 have been used in humans and are expected to be quickly implemented into clinical applications. Only image one molecular marker. Its safety still needs to be further verified in large-scale toxicology studies	TBR: R_0_1-MG-IRDye800 after 24 h (3.5 ± 0.95) was significantly (*P*<0.005) higher compared to control peptide (1.6 ± 0.36), and dye alone (1.4 ± 0.2)	The Pearl Impulse small animal imaging system (LI-COR)
Mucin1	Anti-MUC1 (CT2)-DyLight550/650^27^	In-vitro/in-vivo preclinical probe construction and target validation in mouse model Subcutaneous/orthotopic PDAC mouse model (PANC-1, BxPC-3)	Fluorescence imaging 600 nm	NA NA	NA	Single 30 μg dose	After 7–10 days	It can image pancreatic cancer, accurately locate tumors. Treatment response to MUC1-targeted therapy can also be predicted. Adverse reactions and side effects in humans still need to be explored	TBR (Orthotopic PDAC) Panc-1: 6.70 BxPC-3: 2.39	The Olympus OV100 Small Animal Imaging System
uPAR	ICG-Glu-Glu-AE105^28^	In-vivo preclinical target validation and NIRF-guided surgery in a mouse model	NIRF 800 nm	NA NA	NA	10 nmol	After 15 h	Target uPAR, provide clear tumor signals, locate tumor tissue, and improve the surgical resection rate and negative margin rate. ICG is safe and easy to use. Signal has a short decay time. Due to the heterogeneity of tumors and the expression levels of uPAR. Application may be limited	TBR: (95% CI) PDAC: 3.5 (3.3–3.7) Metastases: 3.4 (3.1–4.0) Identification and removal of additional metastases only on NIRF compared (%) Mice: 4 out of 8 (50%) Metastases: 6/35 (14%)	The FluobeamR800 (Fluoptics, Grenoble, France) robot system da Vinci HD Si (Intuitive Surgical, California, USA) IVIS LuminaXR, a black-box optical camera

CEA, carcinoembryonic antigen; EGFR, epithelial growth factor receptor; ICG, indocyanine green; MB, methylene blue; NIRF, near-infrared fluorescence; PBS, phosphate-buffered saline; PDAC, pancreatic ductal adenocarcinoma; TBR, tumor-to-background ratio; uPAR, urokinase plasmin activator receptor; VEGF, vascular endothelial growth factor.

## Results

### NIRF imaging agents and imaging devices

The classification of NIRF imaging tracers as targeted or nontargeted tracers relies on their specificity. Nontargeted NIRF imaging tracers primarily enhance lesions or structures through the enhanced permeability and retention (EPR) effect or perfusion. On the other hand, targeted tracers are composed of specific molecular probes^29^. To date, the FDA (U.S. Food and Drug Administration) has only approved ICG and MB for intraoperative NIRF imaging.

#### ICG

The most commonly used agent for clinical fluorescence imaging is ICG. Since 1960, ICG has been clinically used for liver function research. ICG has absorption spectra in both the NIR-I region (650–900 nm) and the NIR-II region (1000–1700 nm). Currently, the most commonly used method in clinical practice is to utilize the fluorescence of the NIR-I region, though new types of NIR-II region fluorescence imaging are emerging. The peak spectral absorption of ICG in plasma or blood is 800–810 nm, and the emission peak is 835 nm, which can penetrate tissues up to a depth of 8 mm.

To further improve the imaging quality of NIRF imaging technology, NIR-II biomedical fluorescence imaging has recently been developed. The NIR-II window can be further divided into the NIR-IIa (1000–1400 nm) and NIR-IIb (1500–1700 nm) subregions. As a NIR-I reagent, ICG has an emission shoulder extending into the NIR-II region, with strong fluorescence intensity, and can provide a higher contrast-to-noise ratio for visualizing deep tissues^30^.

The half-life of ICG in the blood is 2.5–3.0 min, and the maximum recommended dose is 2 mg/kg for 12- to 17-year-olds. Patients with a history of iodine allergy are prohibited from using ICG. The incidence of allergic reactions is ~0.05%. In addition, ICG can enter the lymphatic system through the vascular stroma and enter the thoracic duct and blood system through the lymph nodes. In contrast to the short half-life of ICG in the vascular system, ICG can persist in the lymphatic system for more than 24 h, which is beneficial for lymph node assessment.

ICG plays an indispensable role in hepatobiliary surgery, where its main applications include liver function assessment, cholangiography, and determination of the liver resection plane in anatomical liver resection^1^. Moreover, this technique has strong potential for use in detecting occult metastases under laparoscopy. However, there are still many controversies regarding the current application of ICG in treating pancreatic diseases, including the concentration of ICG used, the injection time window, the injection method, and the individualized administration method.

#### MB

MB was first synthesized by the German chemist Heinrich Caro in 1876 and began to be used in colorants, fabric dyes, pigments, photography, and printing. Later, studies found that it has various antibacterial, antiviral, antioxidant, anti-inflammatory, and antimalarial effects^31–36^, so it became widely used in the field of medicine. Currently, it is mostly used as a marker and indicator in medicine and surgery due to its coloring effect^37,38^. It is also used in NIR imaging^2,39^, as its fluorescence spectrum near 700 nm can be detected.

For fluorescence imaging, MB is usually administered intravenously slowly, usually over 3–10 min. When administered intravenously, MB is metabolized in the liver and excreted in the bile and urine within 4–24 h, with a half-life of 5–6.5 h^40–42^.

The advantages of using MB as a fluorescent agent are that it is relatively inexpensive, costing on average 1/5 that of ICG^43^. However, compared with ICG, MB penetrates less tissue but has more autofluorescence in the background because of its hydrophobic nature^7^. Excessive dosage may cause adverse reactions such as nausea, vomiting, headache, and even cardiac arrhythmia; acute hemolytic anemia; elevated pulmonary artery pressure; and impaired lung function. Therefore^40,42,44–46^, it is important to consider dosage and toxicity concerns when using MB. When using MB as a stain, the recommended dose is 1–2 mg/kg, with a maximum of 3 mg/kg^46^. A dose above 7.5 mg/kg/day can cause Heinz body formation in human erythrocytes, so it is contraindicated in patients with Heinz body anemia^47^. MB is also contraindicated in patients with glucose-6-phosphate dehydrogenase (G6PD) deficiency^48^ and in pregnant women due to its potential teratogenicity^42,49^.

Due to the above characteristics, MB is currently used for visualization of lymph node and vital structures, tumor detection, and tissue perfusion assessment, including NIR imaging of the ureters^50^, blood vessels^51^, parathyroid glands^52^, and pancreatic tumors^53^. It can be helpful in breast cancer detection^54^ and sentinel lymph node biopsy^41^.

#### Structures and applications of specific fluorescent probes

Although ICG and MB are good nonspecific fluorescent contrast agents, they are still inadequate for tumor visualization^47,55,56^. Therefore, a recent focus has been to identify and synthesize effective and safe tumor-specific NIRF tracers that can provide enhanced visualization of the primary tumor, metastatic lymph nodes, and distant metastases^4,8^.

NIRF probes are designed to specifically target enzymes, receptors, or antigens that are unique or differentially expressed in tumor cells or in the tumor microenvironment. This targeting effect helps achieve complete tumor resection at the molecular and cellular levels^8^. The identification of targeted molecules can lead to tumor-specific targeting by the operator, suggesting that the application of NIRF probes is a useful strategy in tumor surgery.

**Figure 1:**
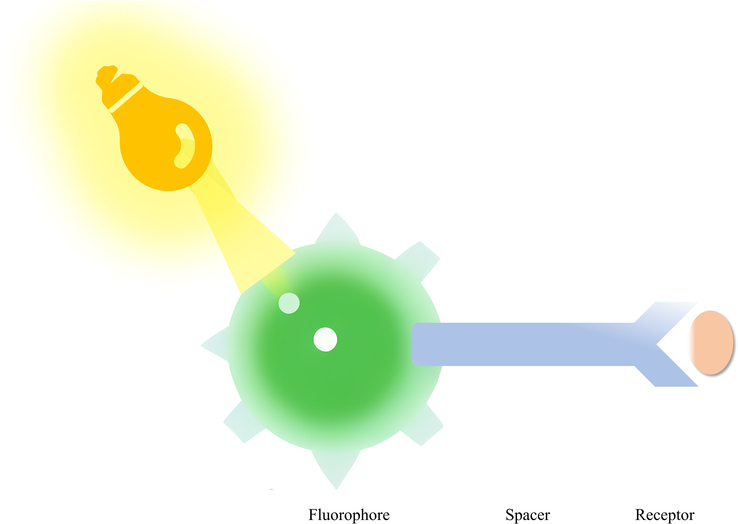
Schematic diagram of fluorescent probe composition. *Note:* The fluorescent probe typically consists of a fluorophore, a spacer, and a receptor. Adapted and reprinted from Ito *et al*.^57^.

Fluorescent probes are usually composed of a fluorescent group (fluorophore), a spacer and a receptor. Among them, the fluorescent group determines the sensitivity of the probe, and the receptor determines the specificity of the probe^57^ (Fig. 1).

NIR-I fluorophores include phlorocyanines, phthalocyanines, porphyrin derivatives, and boron dipyrromethene analogs. NIR-II fluorophores are classified into two major groups, inorganic and organic, based on their chemical structure. Inorganic fluorophores generally possess superior optical properties and adjustable emission wavelengths, but the low biocompatibility and risk of biotoxicity of these materials limit their clinical translation^58,59^. Organic fluorophores have more complete molecular structures, better biocompatibility and lower toxicity and are thus more valuable for clinical translation. The poor photostability of organic fluorophores can lead to photobleaching, which causes poor visualization of the target^60^. The discovery of aggregation-induced emission (AIE) molecules has improved this situation^61^. AIE-type organic molecules can exhibit restricted internal movement and increased light output when aggregated^61,62^, leading to a significant enhancement of fluorescent signals^63,64^.

PDAC recognition targets can be classified into those tumor cells and tumor stroma based on the targeted location^65^. Common tumor cell targets include CEA, EGFR, integrin αvβ6, NTSR1, PSMA, uPA/uPAR, and VEGFR/VEGF-A, while common stromal targets include FAP, MT1-MMP/MMP-14, and integrin αvβ3. Epithelial markers with elevated expression in the extracellular matrix (CDCP-1, EpCAM, and integrin αvβ6) are also potentially effective targets^8^. Only a few of these targets have been evaluated in clinical trials^13,18^.

The information in this article is categorized according to the specific probes that are related to the CEA, EGFR, MUC1, VEGF-A, CA19.9, integrin αvβ3/αvβ5/αvβ6, NTSR1, and uPA/uPAR systems, depending on the recognition group. We then provide an overview of the fluorophores used and their targeting mechanisms, clearance rates, half-lives, recommended injection doses and rates, advantages and disadvantages, and detection devices, with a summary presented in Table 1.

In terms of imaging spectra, NIRF tracers mainly emit light in the NIR-I spectrum. Although the NIR-II spectrum is better for a few reasons, its use is still experimental due to the lack of stability and biocompatibility of the associated fluorophores^66^.

#### NIRF imaging devices

**Figure 2:**
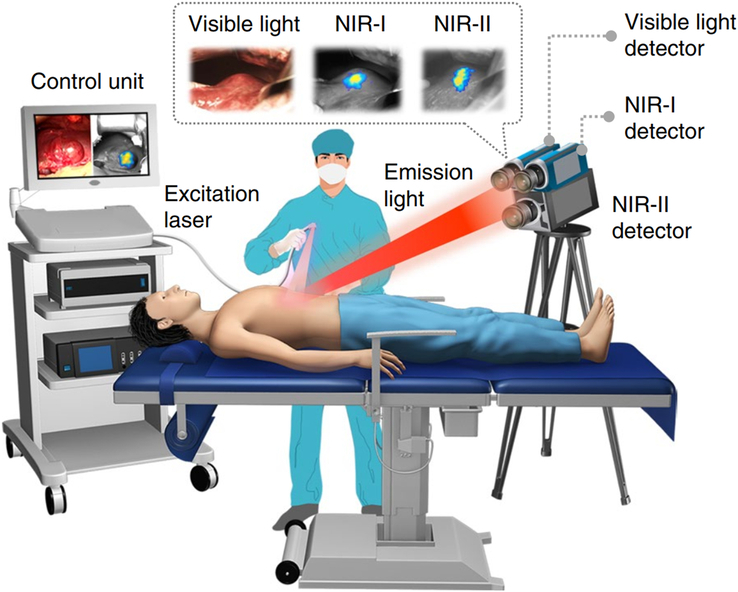
NIRF imaging devices: the components of the imaging process. *Note:* The imaging process involves different components, including an excitation light source, an emission filter, an optical collector, and signal sensors. Adapted and reprinted from Hu *et al*.^30^.

The imaging process involves different components, including an excitation light source, an emission filter, an optical collector, and signal sensors^67^. The excitation light source emits photons of a specific wavelength that pass through the filter and penetrate the tissue. They then reach the fluorescently labeled tracer, which is bound to the target tissue. The fluorophore absorbs these photons, resulting in excitation and enabling imaging of the target tissue. Finally, the NIR imaging system projects the fluorescence image onto the monitor through an optical collector and signal sensors. This imaging process is illustrated in Figure 2.

The commonly used excitation light sources are, in increasing order of spectral bandwidth, laser diodes, light-emitting diodes (LEDs), and filter lamps. Laser diodes and LEDs are more widely used in fluorescence imaging systems than filter lamps due to their advantages of having the lowest background signal, higher output efficiency, and longer lifetime^68^. Filters can filter signals outside the desired spectrum but require a balance of optimized signal brightness and contrast^69^, depending on the fluorescent contrast agent and emitting light source. Optical collectors collect signals into sensors. The field of view depends on the lens design and its focal length. Lenses with shorter focal lengths typically have a wider field of view, making them better for open surgeries. Longer-focal-length lenses are better suited for laparoscopic or robotic surgeries, as they can magnify distant details but offer a smaller field of view. In practice, adjustments need to be made in conjunction with the imaging effect^70,71^. The most commonly used sensors for signal processors in devices are charge-coupled devices (CCDs). Electron-multiplying CCDs (EMCCDs) and intensified CCDs (ICDs) have emerged for NIR imaging to improve signal detection sensitivity but also increase background noise. Complementary metal-oxide-semiconductor (CMOS) cameras and scientific CMOS sensors have become popular fluorescence detection devices due to their high readout rate, small size, low readout noise, and high bit depth^69^.

NIR imaging devices currently come in three forms: handheld, cart-based, and integrated^67,72^. The most commonly used systems are the handheld PDE system and the cart-based miniFLARE system. Other popular systems include the HyperEye Medical System, FLUOBEAM, IC-View, Visualizer, Navigator, FDPM, and SPY. With the advancement of laparoscopic surgical systems and Vinci robotic systems, imaging systems with integrated NIR illumination and detection are growing in popularity. Examples include the INFRARED 800, FIREFLY (da Vinci), and the laparoscopic NIR system. However, the design and performance specifications of these devices can differ significantly, including the fluence rate of the fluorescence excitation source, fluorescence excitation wavelength, CCD camera dynamic range and integration time, working distance, field of view, and detector type. These differences in instrument design can lead to variations in performance, making it difficult to compare results.

Small animal NIR imaging systems are commonly used in preclinical experiments. Popular systems include the NIRF-X-ray dual-mode system, Maestro EX, and in-vivo visible light imaging system (IVIS) by Spectral Instruments Imaging, CRi, and Xenogen^73^. To meet the needs of depth and dynamic real-time observation, new NIR-II region imaging systems have been developed, such as NIR-II region inverted microscopes^74^, confocal microscopes^75,76^, and multiphoton excitation microscopes^77^.

### ICG in pancreatic tumors

#### ICG in PDAC

**Figure 3:**
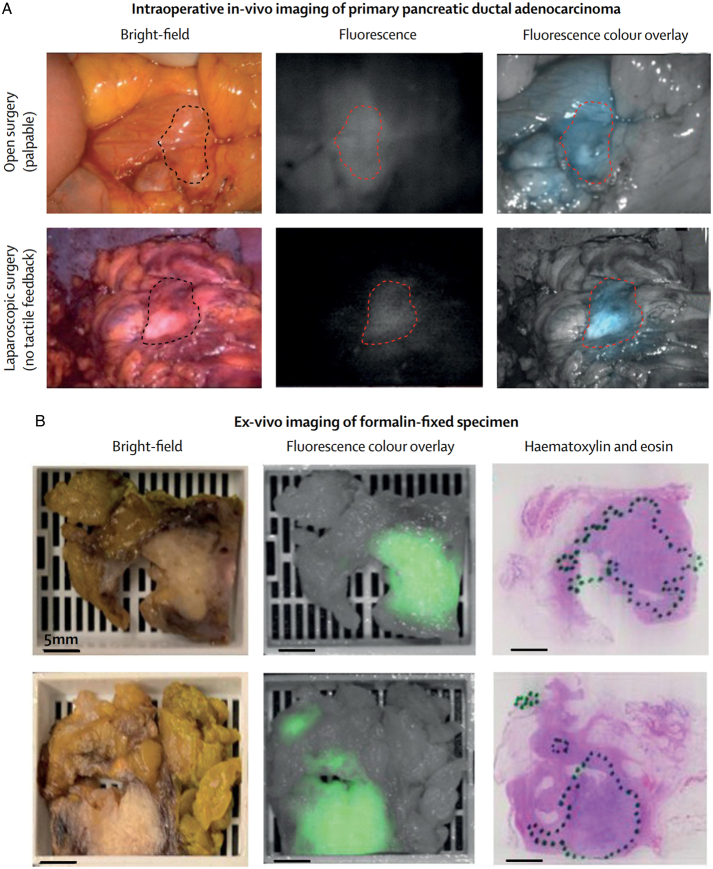
Fluorescence imaging of pancreatic ductal adenocarcinoma. (A) Intraoperative fluorescence imaging in open surgery and laparoscopy for pancreatic ductal adenocarcinoma. (B) Fluorescence distribution of pancreatic ductal adenocarcinoma ex vivo. Adapted and reprinted from Lu G *et al*.^18^

The 5-year survival rate of patients with PDAC is less than 10%^78^. The main advances in the past two decades include the establishment of a preprocessing staging system based on the concept of local resectability/borderline resectability as well as the introduction of combination chemotherapy and targeted therapy. For 15–20% of early pancreatic tumor patients, initial surgical resection is feasible and provides an opportunity for cure, while another 30–35% of patients have locally advanced disease without any detected metastatic lesions. For early pancreatic tumors, despite the considerable progress made by surgical techniques and adjuvant chemotherapy in achieving higher levels of resectability and improving survival outcomes, long-term survival (≥5 years) is still unlikely for most patients^79^. Local recurrence and distant metastasis are key factors shortening postoperative survival. Fluorescence-guided surgery is an intraoperative optical imaging method that can provide surgeons with real-time guidance ton tumor contours, focusing on real-time visualization at the cellular level during surgery^80^. This method is expected to improve the imaging rate of primary tumors and surrounding metastatic lesions as well as surgical accuracy (Fig. 3).

However, compared with that of ICG-guided hepatectomy, the use of ICG-guided navigation in surgery for pancreatic cancer has rarely been reported. Huttman *et al*. explored the use of ICG for fluorescence-guided navigation in surgery for pancreatic cancer and found that in seven of eight (87.5%) patients, the tumor could not be located. The uptake of ICG in the pancreas of tumor patients and healthy patients was equivalent, and no EPR effect was observed^55^. Newton *et al*. explored the application of NIR-II fluorescence imaging technology in pancreatic tumor resection with a clinical trial involving the most enrolled patients for pancreatic tumor fluorescence imaging. In the NIR-II region, ICG had a sensitivity of 100% for detecting invasive malignant pancreatic tumors. All five benign tumors showed no enhancement, for a negative predictive value of 100%. The positive predictive value for the pancreatic neck incision margin was 83.3% (5/6), and the negative predictive value was 100% (7/7). Fluorescence imaging has shown a good correlation with the efficacy of neoadjuvant therapy (with an accuracy of 100%), which helps to determine intraoperative boundaries and make surgical treatment decisions^81^. Matsuki *et al*. injected 3–10 mg of ICG directly into the pancreatic head parenchyma, combined with intestinal rotation technology (a simplified anatomical approach that provides a clear surgical view), to quantify the imaging value of ICG for lymph nodes using NIRF imaging^82^. Among the 10 patients who underwent fluorescence-guided pancreaticoduodenectomy for periampullary tumors, 9 patients exhibited lymph flow from the pancreatic head to the superior mesenteric artery (SMA) through the inferior pancreaticoduodenal artery (PDA) and the first jejunal artery (JA), but not through the second or later arteries, and finally it drained into the SMA and the paraaortic region. The data suggest that the lymphatic pathway of the pancreatic head relates to the SMA through the inferior PDA and the first JA, and removing the pancreatic mesentery along the inferior PDA and the first JA while retaining the second or more distant arteries seems to be the best choice for pancreatoduodenectomy in patients with periampullary malignant tumors. Hirono *et al*.^83^ analyzed the lymphatic drainage pathways in the pancreatic head during Whipple’s operation using ICG fluorescence imaging. ICG was injected into the anterior (*n*=10) or posterior (*n*=10) surface of the pancreas head intraoperatively in 20 patients. Seven major lymphatic drainage pathways were identified under the guidance of ICG fluorescence imaging in real-time, which clarified the extent of lymph node dissection for Whipple’s operation. The lymphatic pathway reaching the left side of the SMA was observed in four patients (20%), while it reached the PA region in 17 patients (85%). The mean time to reach the SMA was longer than that to reach the PA region. Álvarez *et al*.^84^ observed ICG in lymphatic drainage in six patients with adenocarcinoma of the head of the pancreas who underwent open-access duodenopancreatectomy. In all patients, 1 ml of ICG diluted to 10% was given, three patients were infused peritumorally, and three patients were infused intratumorally. The results suggest that drainage and first nodal determination are satisfactory when applied intratumorally. Lymphatic drainage was positive at hepatic artery station 8 in five patients and at the interaortocaval station in one patient in this region.

Li *et al*. conducted murine and human studies of NIR-I/NIR-II imaging of pancreatic tumors using ICG. After the administration of ICG (0.22 mg/kg and 0.50 mg/kg) to nine patients, the tumor tissue exhibited brighter fluorescence than the normal tissue (including tumor margins and normal pancreatic tissue)^10^. ICG-NIRF-II yielded better image quality (a higher tumor-to-background ratio, TBR) than ICG-NIRF-I because its spontaneous fluorescence and light scattering in the NIRF-II region were lower than those in the NIRF-I region.

There are few reports on the application of NIRF for detecting metastasis of pancreatic cancer. Yokoyama *et al*.^85^ evaluated the effectiveness of ICG fluorescence imaging in identifying hepatic micrometastases in pancreatic cancer patients. Forty-nine consecutive patients with pancreatic cancer who underwent surgical intervention were examined. Preoperative clinical images did not reveal any hepatic metastases. Preoperative intravenous injection of ICG was used, and abnormal fluorescent foci in the liver were observed intraoperatively using a NIRF imaging system. Abnormal fluorescent signals with a maximum diameter of at least 1.5 mm were observed in the livers of 13 patients, and histopathological examination confirmed hepatic micrometastases in 8 of these patients (16%). Follow-up within 6 months after surgery showed that seven of the eight patients with pathologically confirmed hepatic micrometastatic carcinoma had obvious hepatic metastatic tumors (88%), while only 4 of the other 41 patients had obvious hepatic metastases (10%). Three of the five patients with fluorescence but without histopathological confirmation had hepatic metastases. Katada *et al*.^86^ reported, in a larger sample, that among 133 pancreatic cancers with no liver metastases detected on preoperative examination, liver micrometastases were detected in 20 cases (15%) intraoperatively, and immunohistochemistry (IHC) further confirmed that these 20 micrometastases all expressed carcinoembryonic antigen (CEA). This suggests that fluorescence imaging can effectively detect liver micrometastases during pancreatic cancer surgery. The investigators indicated that real-time fluorescence imaging may be associated with cholestasis caused by cancer invasion.

**Figure 4:**
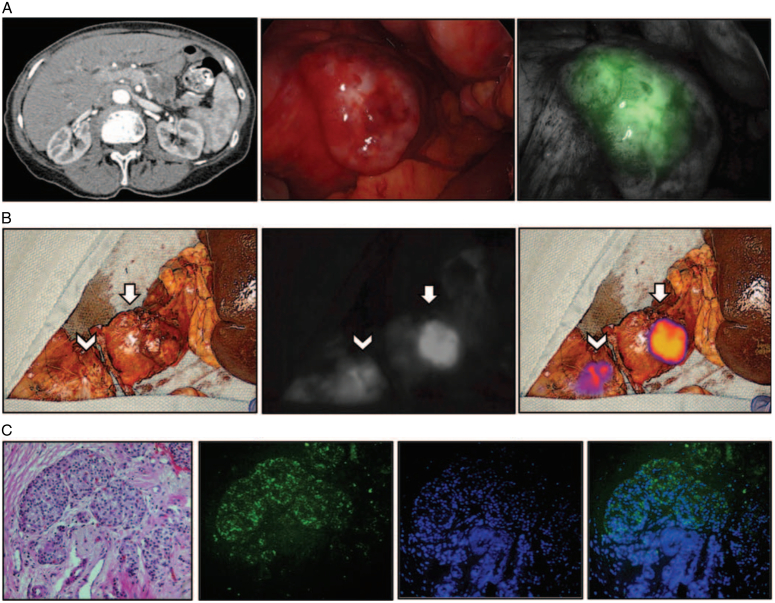
Intraoperative near-infrared (NIR) imaging of pancreatic adenocarcinoma in a patient. (A) Preoperative computed tomography (CT), white light mode and fluorescence mode. (B) Back table white light and NIR images. (C) Hematoxylin and eosin (HE)-staining and indocyanine green (ICG) fluorescence staining of tumors. Adapted and reprinted from Newton *et al.* ^81^.

While intraoperative ultrasound can be used to identify metastatic lesions located deep in the liver, intraoperative fluorescence can be used to identify small lesions that cannot be observed with the naked eye. Combining these two methods is expected to increase the resection rate of primary tumors and small metastatic lesions. Handgraaf *et al*.^87^ found that injecting 10 mg of ICG 1–2 days before surgery, combined with intraoperative fluorescence and ultrasound, could effectively identify small liver and peritoneal metastases during surgery. In their study, 15 patients and 9 patients received ICG doses 1 day and 2 days prior to surgery, respectively. The quality of the laparoscopic near-infrared fluorescence imaging (LFI) was good in 67% (10/15) of the patients who were dosed 1 day and 89% (8/9) of the patients who were dosed 2 days prior to surgery. A futile laparotomy was averted in three patients (12%). This method adequately visualized underlying liver lesions. Oba *et al*.^88^ conducted the SLING trial (Staging Laparoscopy + contrast-enhanced Intraoperative ultrasound + ICG fluorescence imaging) by combining multiple tests for the detection of occult metastases in pancreatic cancer. A total of 31 patients participated in the study, of whom 12 (39%) had occult liver metastases. For patients with liver metastases, ICG-FI best detected lesions in four patients, while CE-IOUS best detected lesions in two patients. Overall, ICG-FI and CE-IOUS complemented each other to improve the detection rate of occult liver metastases. Shirakawa *et al*.^89^ explored the use of ICG combined with fluorescence laparoscopy to evaluate nonmetastatic PDAC patients (0.5 mg/kg) by imaging before performing pancreatectomy or chemotherapy (ClinicalTrials.gov: jRCT1051180076). Hutteman *et al*.^55^ reported that intraoperative visualization may improve the rate of radical resection of pancreatic tumors. Eight patients who underwent pancreaticoduodenectomy received an intravenous injection of 5 or 10 mg of ICG; under the real-time guidance of intraoperative fluorescence imaging, tumors, bile ducts, and pancreatic tissues could be distinguished. However, overall, no significant difference was observed between the tumor and the pancreas. The fluorescence in the common bile duct was clearly visible 10 min after ICG injection. ERCP and cholangiopancreatoscopy are considered standard methods for evaluating and treating pancreatic and biliary duct diseases, providing improved biopsy qualities and direct optical visualization of epithelial lesions. However, white light endoscopy is based on visual examination, which results in a high false-positive rate and a lack of specificity, with limited ability to detect small or early lesions. Glatz *et al*.^90^ applied intraoperative real-time fluorescence cholangiopancreatography for the first time in two patients with pancreaticobiliary diseases, achieving a spatial optical resolution of ~50 mm and detecting an ICG concentration of 17.3 nM (Fig. 4).

Accurately evaluating the response to adjuvant therapy, which is a key step in restaging and determining resectability, remains challenging. Currently, treatment responses are monitored through CT imaging, and radiologists evaluate them using the internationally standardized RECIST 1.1 guidelines^91^. These standards focus on the percent change in tumor size (longest diameter) to determine the treatment response, which is classified as a complete response (CR), partial response (PR), disease progression (PD), or stable disease (SD). Although this method has limited effectiveness in evaluating response, in addition to overestimating tumor size on CT, the value of changes in tumor attenuation in predicting resectability is limited due to the inability to distinguish between treatment-related necrosis and treatment-induced tumor attenuation. In a study of 129 borderline-resectable patients, enhanced CT only showed tumor progression in 1%, while R0 resection was achieved in 74% of patients^92^. Hutteman *et al*.^55^ performed pancreaticoduodenectomy (Whipple procedure) in eight patients with suspected pancreatic cancer and intraoperatively administered ICG intravenously, and the tumor/pancreas fluorescence ratios in the 5 mg group versus the 10 mg group were 0.89±0.25 versus 1.22±0.39, suggesting that ICG fluorescence images did not provide effective tumor demarcation. This may be associated with the low doses of ICG. Newton *et al*.^81^ conducted a prospective clinical trial to evaluate the effectiveness of ICG fluorescence imaging for identifying pancreatic tumors and defining margins. Twenty patients with pancreatic cancer underwent Whipple’s procedure and were given high-dose ICG injections of 2.5–5 mg/kg for 24 h before the procedure, and fluorescence imaging revealed fluorescence signals in 3 out of 8 noninvasive tumors and 12 out of 13 invasive tumors. Of the patients with pancreatic ductal adenocarcinoma, 91.7% had fluorescence, with a tumor background ratio of 4.62±2.95; 12 of the 13 invasive tumors had surgical margin fluorescence consistent with the final pathology; and the probability of positive prediction of pancreatic neck margin fluorescence was 83.3%, with a 100% probability of negative prediction. Intraoperative near-infrared imaging using a second window of ICG had a sensitivity of 100% for surviving invasive malignant tumors. In addition, this study was the first to propose associating fluorescence intensity with the degree of tumor necrosis, further establishing a good correlation with the efficacy of neoadjuvant therapy (poor efficacy: tumor residual >90%, strong fluorescence signal; moderate efficacy: tumor residual 10–90%, moderate fluorescence signal; good efficacy: tumor residual <10%, weak fluorescence signal). It is still unclear whether neoadjuvant chemotherapy affects the clearance rate or tissue absorption of ICG. We believe that the use of fluorescence imaging technology during surgery to identify the activity of pancreatic tumors after neoadjuvant chemotherapy has important prospects for application in pancreatic surgery.

#### ICG in PanNETs

PanNETs are Langerhans cell-derived tumors with an incidence of 6.98 per 1,00000 people^93^. Functionally active tumors include insulinomas, gastrinomas, pancreatic hyperglycemic tumors, and growth-inhibitory tumors. Whereas a small percentage (10%) of patients may have significant clinical syndromes due to an increase in hormones secreted by the cells involved, most (90%) tumors secrete only small amounts of active peptides or no hormones at all^94^. Due to the small size and relatively benign nature of localized PanNETs, preoperative and intraoperative localization are crucial for oncologic lymphadenectomy, for achieving clear resection margins, and for preserving pancreatic function^95^.

**Figure 5:**
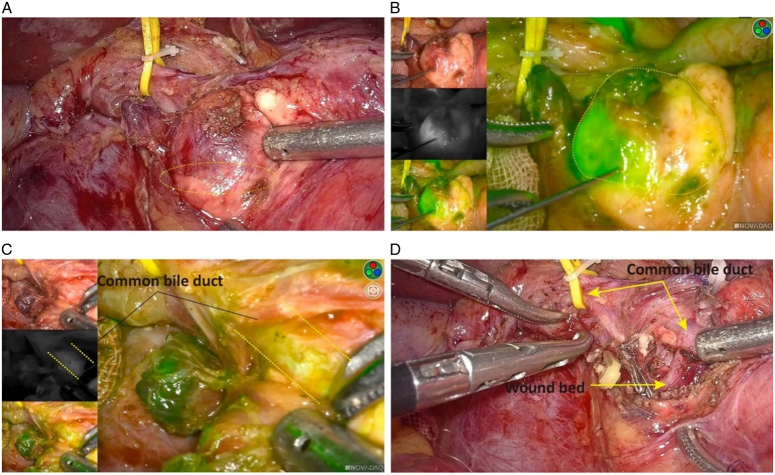
Insulinoma enucleation process, subcapsular indocyanine green (ICG) injection, identification of the biliary tract, and postoperative wound bed. (A) No clear boundary of the tumor and common bile duct can be observed (yellow-dashed ellipse). (B) ICG (0.025 mg/ml) was laparoscopically injected under the capsule of the tumor (the yellow-dashed circle refers to the range of clear boundaries presented after local injection of ICG). (C) The common bile duct was detected based on fluorescence. (D) After enucleation, the wound bed and common bile duct were inspected. Adapted and reprinted from Tao *et al*.^100^.

The currently reported laparoscopic surgery rate for PanNETs is 5.5%, with a laparoscopic removal rate of 65%^96^. One of the most challenging aspects of surgical planning is accurate tumor localization. Considering small PanNETs, the reported preoperative and intraoperative detection rates are as high as 92%^97^; even with the use of palpation and intraoperative ultrasound, the detection rate decreases to 50% for tumors smaller than 1 cm^98^. Even in centers with a high surgical volume, the intraoperative detection rate cannot reach 100%. Accurately removing tumors while preserving the integrity of important surrounding tissues, such as the main pancreatic duct, peripancreatic blood vessels, and mesenteric blood vessels, is highly important for patient prognosis. Claudio Bassi performed laparoscopic pancreatic surgery for PanNETs in 10 patients^11^. During surgery, the patients were injected with ICG (25 mg, divided into five injections of 5 mg), and NIR imaging was used to identify all 10 PanNETs. Nine patients (90%) underwent laparoscopic distal pancreatectomy and splenectomy, and one patient (10%) underwent laparoscopic tumor removal. Eight nonfunctional PanNETs (80%) and two insulinomas (20%) were found in the final pathology examination. Nine-tenths (90%) of PanNETs were detected after the second ICG injection. The average latency was 80 seconds, and the average visible time was 220 s. The peak tumor visualization was reached 20 min after the last injection. Post hoc analysis of the fluorescence signals confirmed these findings, with an average signal-to-background ratio (SBR) of 7.7 (*P*=0.001). This study indicated that fluorescence imaging technology based on ICG could play an important role in the localization and surgical resection of PanNETs. Tao *et al*.^99^ used intraoperative fluorescence imaging technology to detect small liver metastases in pancreatic cancer patients with negative preoperative imaging findings; in 49 patients with pancreatic cancer who underwent surgical intervention, the preoperative imaging did not show liver metastases. An intravenous injection of 25 mg of ICG 24 h before surgery helped visualize liver micrometastases (≥1.5 mm) in 13 patients. Due to the presence of capsules in most insulinomas, Fang’s team found that dynamic ICG perfusion (preoperative intravenous injection combined with intraoperative pseudocapsule injection of ICG) combined with intraoperative ultrasound (IOUS) could shorten the localization time and improve the success rate of radical resection for some occult insulinomas. Injecting ICG under the pseudocapsule may provide a new approach for exploring ICG fluorescence imaging methods suitable for benign tumors^100^. Constantinescu *et al*.^101^ reported a patient with a pancreatic head tumor in whom they used NIR to detect synchronous pancreatic tumors and potential secondary lymph node or liver involvement. After the patient was diagnosed with insulinoma of the pancreatic head, NIR imaging was performed on the tumor mass, and pylorus-preserving pancreaticoduodenectomy was performed. Although no other synchronous lesions, secondary lymph node involvement, or liver metastases were detected intraoperatively, the investigators concluded that the combination of NIR and ICG helped to accurately detect pancreatic neuroendocrine tumors and the other aforementioned lesions. This is because the patient’s 30-day postoperative review revealed no signs of short-term recurrence or metastasis (Fig. 5).

### MB in pancreatic tumors

**Figure 6:**
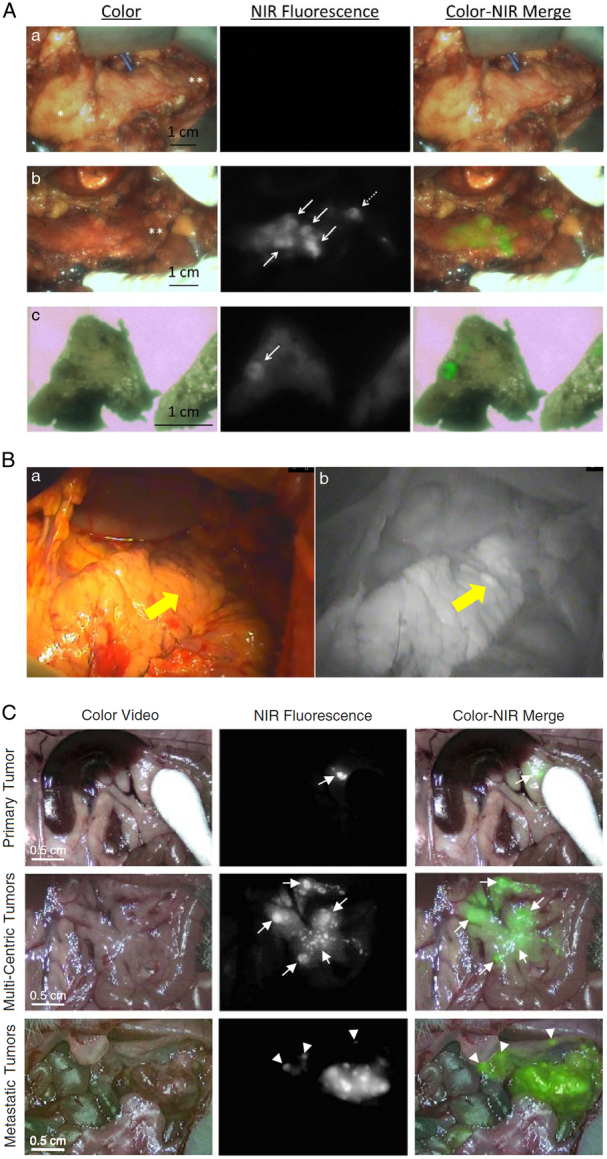
(A) Intraoperative and ex-vivo near-infrared region (NIR) fluorescence imaging of the pancreas. (a) Prior to intravenous administration of methylene blue (MB). No autofluorescence was observed (exposure time, 50 ms). (b) Five minutes after the start of the infusion of MB. Multiple lesions in the head, body, and tail of the pancreas are fluorescent (exposure time, 50 ms). (c) Ex vivo. Faint fluorescence signals are still visible (exposure time, 200 ms) 3 days after resection and formalin fixation. White arrows: lesions suspect for PNETs. *Head of the pancreas. **Tail of the pancreas. Dashed arrow: this suspect lesion was stained for chromogranin and synaptophysin (Fig. 3). Adapted and reprinted from Handgraaf *et al*.^105^. (B) Methylene blue (MB) fluorescence imaging to visualize the pancreatic neuroendocrine neoplasm (NEN). (a) Gross appearance of the pancreas. The NEN (arrow) was unclear on the pancreatic surface. (b) At 20 s after MB fluorescence imaging. Adapted and reprinted from Fukuda *et al*.^53^. (C) NIR fluorescence imaging of insulinoma using intravenously injected MB. Insulinoma-bearing NOD/ShiLt-Tg(RipTAg)1Lt/J mice were imaged 15 min after intravenous bolus injection of 1.5 mg/kg MB. Shown are color video (left), 700 nm NIR fluorescence (middle), and a pseudocolored (lime green) merge of the two (right). NIR fluorescence images were acquired with a 150 ms exposure time and displayed with identical normalizations. Shown are animals bearing a single primary tumor (arrow) in the pancreas (top row), multicentric pancreatic tumors (arrows; middle row), and metastatic tumors (arrowheads; bottom row). Adapted and reprinted from Winer *et al*.^103^

MB is also commonly used as a fluorescent dye. MB can be excited by a laser at 680 nm and visualized at ~710 nm. MB is commonly used to locate the parathyroid gland and ureters during surgery to prevent accidental damage during the operation. It is also used for edge detection during breast-conserving^54,102^ and pancreatic parenchyma-preserving therapy for breast cancer and pancreatic tumors. Winer *et al*.^103^ rapidly injected MB into wild-type rats, pigs, and transgenic mice harboring insulinomas at doses ranging from 0.25 to 2 mg/kg, and its utility in intraoperative fluorescence imaging of insulinomas was explored. When the dose is ≥1 mg/kg, MB will accumulate in certain normal tissues, such as the pancreas, and maintain NIR fluorescence for up to 1 h at an SBR ≥1.6. Due to the higher uptake of MB by insulinoma tissue than by normal pancreatic tissue, the insulinoma-to-pancreas signal ratio was 3.7, and the insulinoma-to-muscle signal ratio was 16.2. MB allows for high sensitivity and real-time localization of primary, multicentric, and metastatic insulinomas, as well as differentiation between tumors, normal pancreatic tissue, and other abdominal structures. Bert A Bonsing and colleagues used MB (1 mg/kg) for intraoperative imaging in a patient with an isolated pancreatic fibroma. Five minutes after the intravenous injection of MB, the tumor background ratio reached 3 and remained stable for the next 15 min without any adverse reactions. They identified the solitary fibrous tumor of the pancreas using NIR fluorescence and MB during surgery^104^. Multiple endocrine tumor type 1 syndrome is characterized by pancreatic neuroendocrine lesions that may undergo malignant transformation. After intravenous administration, MB can accumulate in neuroendocrine lesions. Handgraaf *et al*.^105^ used MB combined with intraoperative NIRF imaging as a safe and relatively simple technique to improve the evaluation of neuroendocrine lesions during surgery (Fig. 6).

Fukuda *et al*.^53^ applied MB for fluorescence visualization in 18 surgeries on the liver and pancreas. The fluorescence intensities of blood vessels, tumors, liver, and intestines were measured. Successful visualization of the target object by MB fluorescence imaging was achieved in 17 (94%) cases, including 100% of neuroendocrine tumors (4 tumors) and peripheral pancreatic vessels (*n*=13), and there were no complications related to MB. Administering MB and applying fluorescence imaging with MB can be used to visualize blood vessels and pancreatic neuroendocrine neoplasms. This study also demonstrated that MB is safe for Japanese patients without methemoglobinemia.

### Other drug–fluorescent conjugates in pancreatic tumors

**Figure 7:**
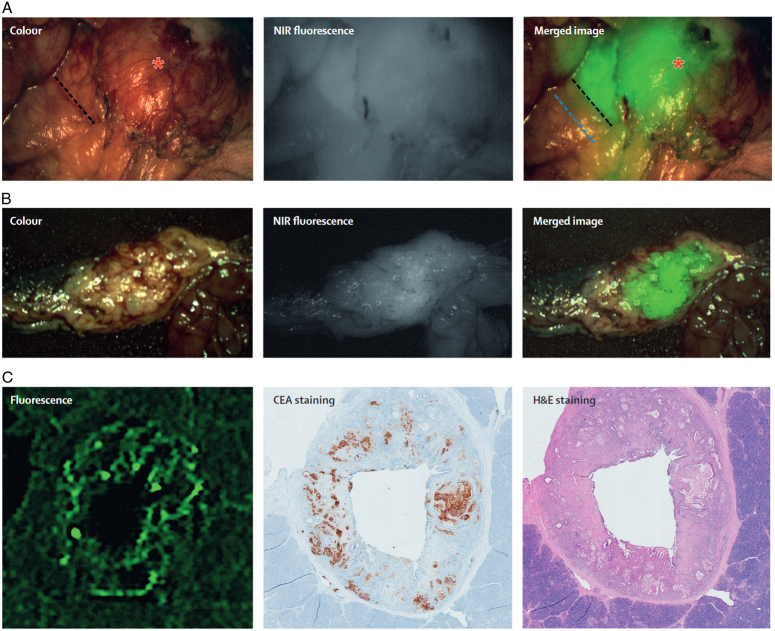
Example of intraoperative molecular fluorescence imaging during pancreatic surgery. (A) Intraoperative fluorescence imaging of a pancreatic tumor, which could be visualized using a monoclonal antibody for carcinoembryonic antigen conjugated to a 700 nm fluorophore (SGM-101). On the basis of the image, surgeons decided to move the planned resection line (black dashed line: proposed resection line based on visual inspection and palpation; blue dashed line: proposed resection line based on fluorescence). (B) Ex-vivo analysis of a resected pancreatic tumor. (C) Histopathological analysis of a tumor slice of a primary pancreatic tumor showing fluorescence, carcinoembryonic antigen expression, and microscopically localization of tumor cells. Adapted and reprinted from Hoogstins *et al*.^13^ and Hernot *et al*.^9^.

Many tumor-specific imaging agents, including integrin αvβ6, carcinoembryonic antigen (CEA), epithelial growth factor receptor (EGFR), and urokinase plasmin activator receptor (uPAR), seem to have high potential for PDAC-specific fluorescence imaging^106^. As a glycoprotein produced by gastrointestinal tissue, CEA is overexpressed in many types of cancer, including PDAC. Hoogstins *et al*.^13^ reported that SGM-101 (molecular weight, 148.6 kDa) specifically accumulates in primary tumors expressing CEA, as well as in peritoneal and liver metastases, enabling real-time intraoperative fluorescence imaging. The average TBR of primary tumors was 1.6, and that of metastatic lesions was 1.7. However, visualization of the lymph nodes by contrast is limited because the lymph nodes may be covered with blood or tissue. Lu *et al*.^18^ were the first to use panitumumab-IRDye800CW for the clinical detection of PDAC. As panitumumab-IRDye800CW is effective in pancreatic cancer surgery, they expected it to provide enhanced visualization of surgical margins, metastatic lymph nodes, and distant metastases. A total of 11 patients with PDAC were included in their study. Patients received panitumumab-IRDye800CW in three dose groups: 25 mg, 50 mg, and 75 mg. The optimal dose determined by the mean TBR of the primary tumors was 50 mg, which enabled clear observation of them, with a TBR of 4.0 (SD=0.6). Intraoperatively, near-infrared fluorescence imaging was used to visualize the primary tumor, metastatic lymph nodes, and small (<2 mm) peritoneal metastases. Based on the fluorescence signal, 26 suspicious lymph nodes were removed intraoperatively in 11 patients. Three (12%) of these lymph nodes were found to contain metastatic adenocarcinoma. Sixty-seven (17%) of the additional 358 lymph nodes in the isolated pancreatectomy specimens were diagnosed as metastatic adenocarcinoma. The mean fluorescence intensity of ex-vivo images of metastatic lymph nodes was significantly higher than that of benign lymph nodes (*P*≤0.0001); although a high intraoperative false-positive rate was observed, the increased sensitivity avoided missing metastatic lymph nodes from the point of view of complete intraoperative clearance. Tummers *et al*.^107^ evaluated the safety and feasibility of multimodal molecular imaging of primary and metastatic tumors during surgery using cetuximab-IRDye800. This study included seven patients with resectable pancreatic masses and a suspected diagnosis of PDAC. Fluorescence imaging successfully revealed the tumor, with an average fluorescence intensity inside the tumor (0.09 ± 0.06) significantly higher than that in the surrounding normal pancreatic tissue (0.02 ± 0.01) and tissue affected by pancreatitis (0.04 ± 0.01, *P*<0.001), with a sensitivity of 96.1% and a specificity of 67.0%. The average photoacoustic signal at the tumor site was 3.7 times that of the surrounding tissue. PanNETs, such as insulinomas, are difficult to locate during surgery, and complete resection of these tumors is key to achieving a cure. In addition, multimodal molecular imaging has shown good results in detecting lymph node metastasis and distant metastasis. In this study, tumor-specific imaging was successfully performed in patients with pancreatic cancer using a near-infrared fluorescently labeled antibody to detect primary tumors, tumor-bearing lymph nodes, and distant metastases. The mean fluorescence signal-to-background ratio of positive lymph nodes was 6.3 ± 0.82. The mean fluorescence signals were significantly different between tumor-bearing lymph nodes (*n*=29) and tumor-negative lymph nodes (*n*=78) (0.06 ± 0.01 vs. 0.02 ± 0.002, *P*<0.001). Liver metastases could also be detected by negative contrast. Taken together, the results of this study demonstrate that multimodal molecular imaging can be successfully applied to surgically resect pancreatic cancer to identify primary tumors, positive lymph nodes, and distant metastases. Tummers *et al*.^108^ went on to evaluate the visualization of peritumoral lymph nodes using the cetuximab-IRDye800 fluorescent imaging tracer (targeting EGFR) in patients after pancreatic cancer surgery in a prospective study involving dose escalation of fluorescent probes. The results showed better detection of metastatic lymph nodes in the low-dose (50 mg) group, with a specificity of 78%. More importantly, occult lymph nodes (<5 mm in diameter) of the tumor could be better detected with a sensitivity of 88% (15/17 lymph nodes). These findings show the great potential of targeted fluorescent reagents combined with fluorescence imaging to detect lymph node metastasis. Hoogstins *et al*.^13^ evaluated the application of a novel fluorescently labeled anti-CEA antibody, SGM-101, in intraoperative fluorescence tumor imaging. Twelve patients received an intravenous injection of 5, 7.5, or 10 mg of SGM-101 at least 48 h before surgery for pancreatic cancer, and the surgical area was imaged using an NIR imaging system. The results showed that SGM-101 specifically accumulated in primary tumor, peritoneal, and liver tissues with high expression of CEA. The average TBR was 1.6 for primary tumors and 1.7 for metastatic lesions. Pancreatic mesenteric lymph node dissection is an important component of pancreaticoduodenectomy, but due to the complex anatomical structure of the pancreatic mesangial lymphatic pathway, the optimal range for lymph node resection is still unclear (Fig. 7).

Although no tumor-specific imaging agent has been reported for PDAC after neoadjuvant treatment assessment, relevant and promising molecular imaging targets have been demonstrated to differentiate tumor tissue, normal tissue, and inflammatory pancreatic lesions^109^. Investigators collected resection specimens from patients after neoadjuvant FOLFIRINOX treatment, including PDAC (*n*=32), tumor-associated pancreatitis (TAP) and therapy-induced fibrosis (TIF) (*n*=15), normal pancreatic parenchyma (NPP) (*n*=32), and tumor-positive (*n*=24) and tumor-negative lymph nodes (*n*=56). The expression of 8 biomarkers, integrin αvβ6, CEACAM5, mesothelin, PSMA, uPAR, FAP, ITGA5, and EGFR, was evaluated using IHC to assess the contrast of these targets. The results showed that integrin αvβ6, CEACAM5, mesothelin, and prostate-specific membrane antigen (PSMA) exhibited significantly higher H-scores in PDAC tissue than in NPP and TAP tissue (*P*<0.001). Further analysis of the H-scores revealed a tumor‑to‑normal ratio (TNR) of 4.1 for integrin αvβ6, 28.5 for CEACAM5, 25.5 for mesothelin, and 99.4 for PSMA. IHC staining revealed 24 true-positive (TP) and 56 true-negative (TN) lymph nodes when stained for integrin αvβ6, 20 TP and 60 TN lymph nodes when stained for CEACAM5, 16 TP and 63 TN lymph nodes when stained for mesothelin, 15 TP and 63 TN lymph nodes when stained for mesothelin, and 15 TP and 24 TN lymph nodes when stained for PSMA. This approach resulted in both a sensitivity and a specificity of 100% for integrin αvβ6 and values of 83% and 100%, respectively, for CEACAM5. The study also found that integrin αvβ6, CEACAM5, mesothelin, and PSMA were expressed significantly higher in PDAC than in TAP and NPP. Metastatic lymph nodes could also be accurately detected based on the integrin αvβ6 and CEACAM5 levels.

### Current challenges in pancreatic fluorescence-guided surgical navigation

Current challenges mainly arise from the blocking effect of fluorescent imaging agents, imaging equipment, and the fibrous matrix around pancreatic tumors. Due to the limited blood supply to the pancreatic parenchyma, systemic doses of ICG are not likely to accumulate much in tumors. The presence of a dense fibrous matrix around pancreatic tumors is also an important factor in blocking drug entry into tumors. In addition, ICG accumulates in tumors due to the EPR effect, resulting in limited targeting efficiency. The identification of pre-ICG fluorescence in pancreatic tumors is limited to case series or relatively small single-center cohort studies. Systematic reviews and meta-analyses have shown that intraoperative ICG light can guide surgeons in identifying pancreatic lesions with a high accuracy of 81.3%^110^. These results may be influenced by device sensitivity and tumor false negatives, with different fluorescence intensities depending on the depth of the nodules in the pancreatic parenchyma.

There are still limitations in the clinical use of special fluorescent contrast agents. This is due to the potential biosafety risks associated with some NIR-II fluorophores, including heavy metal leakage and long-term retention in the body. Additionally, for fluorescent contrast agents that are clinically safe, standards and specifications for their human use still need to be explored in clinical studies. Thus, although the imaging depth of the NIR-II region has been improved (~15 mm), it is still insufficient for deep pancreatic tumor imaging. Finally, no universally available and affordable NIR imaging systems, especially for the NIR-II region, are available to gain experience and draw meaningful conclusions.

In addition to the display of the tumor itself, due to the biological characteristics of pancreatic cancer, the imaging of metastatic lymph nodes and distant metastases (especially liver metastases and peritoneal metastases) and the determination of surgical margins after neoadjuvant surgery via NIRF have become increasingly important in assisting surgeons with surgical decision making. Current studies suggest that ICG/MB or NIRF combined with specific contrast agents can significantly improve the detection rate of metastatic lymph nodes and liver metastases and have been applied in the evaluation of the effect of neoadjuvant therapy and the judgment of intraoperative borders. However, these studies had small samples, were focused on intraoperative visualization and short-term prognosis, and lacked long-term patient survival data. Larger, multicenter, high-quality clinical studies are needed to validate the clinical value of NIRF and to clarify the long-term survival benefit of intraoperative NIRF.

## Discussion and future directions

Preoperative evaluation of pancreatic tumors and intraoperative identification of tumors are often challenging. For intraoperative ultrasound, adhesions caused by previous surgery, inflammation, chronic pancreatitis, or neoadjuvant chemotherapy are particularly challenging and limiting. ICG fluorescence imaging is increasingly being applied in various surgical fields due to its low cost, simplicity, and few side effects. It can provide valuable intraoperative information for surgeons, especially when inputs are limited or missing, such as during surgery performed via laparoscopic or robotic technology. Surgical navigation via fluorescence can be used to enhance the visualization of anatomical structures and boundaries, evaluate organ perfusion in real-time, identify tumors, and detect invisible metastatic tumors.

According to the fluorescence expert consensus published in 2023^111^, more than 80% of experts agreed that the main application prospects of ICG in pancreatic surgery lie in the following: (1) visualizing the anatomical structure of extrahepatic bile ducts through fluorescence during the dissection of the lateral margin of the SMA to avoid damage; and (2) detecting and accurately locating metastatic lesions, determining accurate surgical margins, and visualizing surrounding structures such as bile ducts and lymph nodes. A total of 75–80% of the experts believe that fluorescence combined with other techniques, such as intraoperative ultrasound, can be used to accurately locate lesions, visualize vascular structures (such as the SMA or SMV), determine tumor tissue activity, and determine the vitality of anastomoses and surrounding organs. In this expert consensus, 50% of the experts had been involved in pancreatic cancer surgery for more than 10 years, but 66.7% of the experts had been involved in fluorescence-guided navigation for pancreatic tumor surgery for less than 5 years, indicating that fluorescence-guided navigation for pancreatic tumor surgery is a new surgical method with much room for development and improvement. This also means that new demands will be placed on the surgeon’s operational skills and knowledge base. At present, research evidence on the role of ICG fluorescence in identifying pancreatic tumors is limited to case series or relatively small, single-center cohort studies. We put forth the following: (1) There is a need for more reports on the application of ICG in pancreatic surgery, including drug delivery strategies and injection time windows. (2) Standardized processes and individualized fluorescence parameters for tumor imaging and vascular imaging need to be explored. (3) Combinations of ICG fluorescence imaging with 3D visualization and other intraoperative real-time imaging methods, such as intraoperative ultrasound, virtual reality (VR), augmented reality (AR), and mixed reality (MR), should be studied.

For the application of specific fluorescent contrast agents in pancreatic tumors, which are currently in a stage of rapid growth, future directions include the following: (1) NIR-II fluorescent contrast agents with high sensitivity, specificity, high resolution, and effective penetration that can be used in the human body should be developed. (2) NIR-II fluorescence endoscopic devices with high spatial resolution should also be developed. (3) There is a need for a more complete understanding of the impact of fluorescence-guided navigation in surgery, heterogeneity of fluorescence technologies, accuracy of quantitative evaluation, and standardization of fluorescence instruments.

In addition to the development of specific fluorescent contrast agents and imaging systems, combined intraoperative surgical navigation and tumor imaging based on the application of multiple techniques are also future development directions. For example, multispectral imaging technology^30^, multimodal real-time image fusion technology^112^, artificial intelligence technology^113^, and other visual information-based technologies have been applied in the fields of optics, liver surgery, intelligent diagnosis, and treatment but have not yet been applied in pancreatic tumor surgery. Therefore, establishing a multidisciplinary crossover model, cultivating composite medical–physical–chemical–industrial talent, strengthening collaboration between teams, establishing thorough databases, and strengthening collaborations with industry and academic partners will promote the application of these technologies to surgery.

## Conclusion

The application of fluorescence imaging technology in pancreatic surgery is safe and effective and is expected to bring new hope to the field of pancreatic surgery. Due to incomplete process standardization and interindividual differences, more research is still needed on the concentration and injection time window of ICG/MB. Expanding and optimizing the application scope of ICG/MB in pancreatic surgery will contribute to improvements in tumor resection, organ protection, and patient prognosis.

While specific fluorescent contrast agents are gradually entering clinical application, there is still a need to solve the problems of biological toxicity, imaging effects, imaging equipment costs, and standardized use processes.

In the future, as fluorescence technology advances, we believe that surgeons will more accurately identify tumor boundaries during surgery with the help of clear fluorescence images, more often completely resect residual tumor lesions, maximize the survival period, and improve patient quality of life.

## Ethical approval

This is a retrospective review that does not relate to ethical problems.

## Consent

This is a retrospective review that does not violate ethical regulations.

## Source of funding

This work was supported by the Natural Science Foundation of Hunan Province (2022JJ40216/2024JJ9479), the General funded project of Hunan Provincial Health Commission (B202309046427/R2023096/W20242019).

## Author contribution

W.C., N.Z., K.C., and X.T.: study concept or design; K.C.: data collection and analysis; K.C. and X.T.: writing the paper; W.C.,N.Z.: revised the manuscript.

## Conflicts of interest disclosure

The authors declare no conflicts of interest.

## Data availability statement

The data used to support the findings of this study are available from the corresponding author upon request.

## Provenance and peer review

Not commissioned, externally peer-reviewed.
